# An Unusual Presentation of Merkel Cell Carcinoma in a HIV Patient: A Case Report and Literature Review

**DOI:** 10.1177/2324709619836695

**Published:** 2019-04-02

**Authors:** Preethi Ramachandran, Burak Erdinc, Vladimir Gotlieb

**Affiliations:** 1 Brookdale University Hospitals and Medical Center, New York, NY, USA

**Keywords:** Merkel cell cancer, metastatic disease, subcutaneous nodule, HIV

## Abstract

Merkel cell carcinoma (MCC) is a rare, rapidly growing, aggressive neuroendocrine skin cancer that generally arises on sun-exposed areas of body such as head, neck, upper limbs, and shoulders of people with light complexity. Typically, MCC presents as shiny, flesh-colored or bluish-red, intracutaneous nodule, possibly with ulceration or crusting. In most of the cases, there is an association with Merkel cell polyomavirus. Even though these are very aggressive tumors, early detection and treatment has always given favorable outcome. There seems to be no consensus in definite prognostic markers, and advanced stages have the worst outcome even with treatment. There has been a recent trend in using PD-I/PD-L1 target therapy rather than chemotherapy in these cancers and have shown to improve survival by many months. In this article, we report a very unusual presentation of MCC first found on left frontoparietal skull as an 8-cm diameter fixed, subcutaneous mass without any typical features of MCC and was found to have metastatic spread to lung and liver. The patient was treated with palliative radiotherapy to brain and chemotherapy with cisplatin/etoposide with addition of immunotherapy later.

## Introduction

Merkel cell carcinoma (MCC) is a rare, aggressive skin cancer that originates from neuroendocrine cells, predominantly affecting older adults with light skin. However, it may present at an earlier age in immunocompromised patients such as organ transplant recipients, HIV-infected individuals, and those with B-cell lymphoid malignancies. Characteristically, MCC tumors are rapidly growing, painless, firm, nontender, flesh-colored or bluish-red, cutaneous nodules. They are classically found on sun-exposed areas. On immunohistochemical studies, MCC cells express both epithelial markers (AE1/AE3, CAM 5.2, pancytokeratin, epithelial membrane antigen, and Ber-EP4) and also neuroendocrine markers (chromogranin, synaptophysin, calcitonin, vasoactive intestinal peptide, and somatostatin receptor). It can be differentiated from other poorly differentiated, round, blue cell tumors by being stained with CK20 and CK5/6.

Local disease is treated with excision of the primary lesion with or without adjuvant radiotherapy (RT) and immunotherapeutic agents such as avelumab, pembrolizumab, nivolumab, or systemic chemotherapy with platinum (cisplatin, carboplatin) plus etoposide commonly used for metastatic disease. Herein, we describe a case of MCC with unusual first presentation of metastatic disease to the lung and liver and with primary lesion in the skin presenting with different clinical characteristics not described in literature previously.

## Case Report

**Figure 1.**
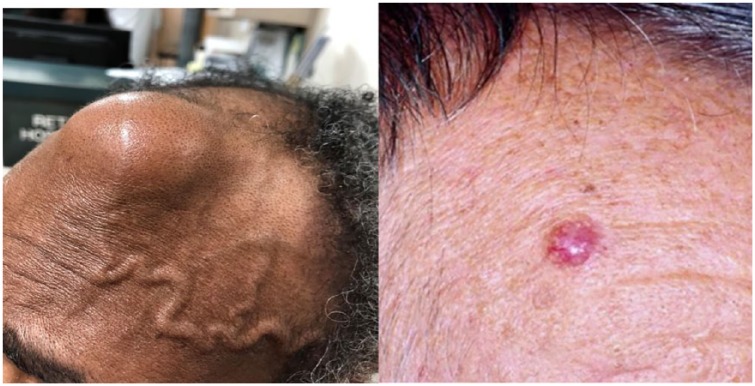
Presentation of our case (left) versus classical picture (right).

**Figure 2.**
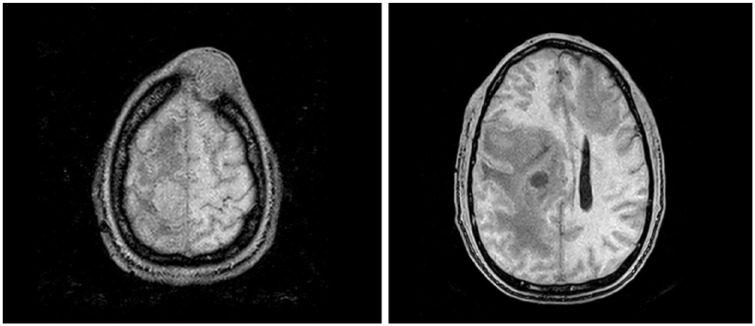
Magnetic resonance imaging showing the primary scalp lesion and the metastatic brain lesions.

A 51-year-old male with past medical history of HIV with CD4 count of 32/mm^3^ presented to the emergency department with a chief complaint of left-sided weakness and altered mental status. He was a poor historian and was falling asleep intermittently during interviewing. Further history from family revealed that he had been diagnosed with HIV for more than 5 years and has been very noncompliant with treatments. On physical examination, the patient’s vital signs were significant only for elevated blood pressure of 150/92 mm Hg. He was noted to have an 8-cm fixed, subcutaneous mass on left frontoparietal skull. The rest of dermatologic examination revealed intact skin without erythema or ulceration. Laboratory investigation was insignificant except for mild leukocytosis (10.7 × 10^9^/L) and neutrophilia (6.4 × 10^9^/L). Computed tomography scan of head without contrast revealed no intracranial hemorrhage; however, multiple masses were noted including a 2.8-cm right superior frontal intra-axial hyperdense mass with an adjacent mixed density 2.7-cm right frontal mass, a 1.6-cm right frontal nodule, a 7-mm right frontal hypodense nodule and a 1.3-cm left frontal nodule. These lesions were associated with marked surrounding infiltrative versus vasogenic edema, which were suspicious for malignancy. In addition, a left frontal infiltrative osseous mass with overlying soft tissue swelling was noted, compatible with malignancy. Subsequent magnetic resonance imaging of the brain showed a 2.5-cm destructive bone lesion in the left frontal skull with a large soft tissue mass in the left frontal scalp and multiple enhancing masses in both cerebral hemispheres measuring up to 2.8 cm in diameter with surrounding edema consistent with metastatic disease to the brain and skull (Figures 1 and 2).

**Figure 3.**
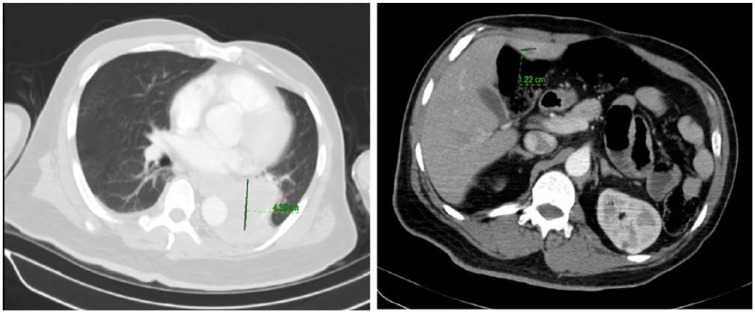
Computed tomography demonstrating large hilar lung mass and liver nodules.

Chest X-ray showed 4.3-cm left hilar mass and subsequent computed tomography of the chest/abdomen/pelvis revealed a 6.2 × 3.8 cm lobulated mass on left hilar region (Figure 3), bilateral pulmonary and liver nodules as well as a pulmonary embolus of right lower lobe pulmonary artery.

Scalp biopsy of the frontal mass demonstrated an extensively necrotic tumor with nested architecture with high NC (nucleus-to-cytoplasm) ratio and indistinct nucleoli. There were many apoptotic bodies and readily identifiable mitotic figures. Tumor cells were positive for CK20, chromogranin A, neuron-specific enolase, and Ki 67—100%, and negative for CK7, thus confirming the diagnosis of MCC. Since he presented with a large hilar mass, which is unusual for metastatic presentation, a biopsy was performed on the hilar mass, which also confirmed that specific markers for MCC favoring metastatic disease rather than primary lung lesion.

The clinical characteristics of the scalp lesion and the large hilar metastatic lung lesion were very unusual presentations and had not been described previously in MCC, which makes this case unique. He was started immediately on central nervous system radiation due to presentation with altered mental status. Immunotherapy (avelumab) was planned, but in the meantime, he underwent 1 cycle of chemotherapy with carboplatin/etoposide until avelumab was available. But within a week of starting treatment, the patient deteriorated with disease progression and died due to respiratory failure.

## Discussion

Merkel cell carcinoma is an uncommon, aggressive primary neuroendocrine skin cancer that predominantly affects older adults with light skin and with a mean age at diagnosis of 76 years for women and 74 years for men.^1,2^ Nevertheless, MCC may occur at an earlier age, and more commonly, in patients with HIV, other immunocompromised states, and those with other active malignancies.^3-9^

Over the past decades, MCC incidence has risen with an annual incidence rate rising from 0.5 cases per 100 000 in 2000 (95% confidence interval = 0.4-0.5) to 0.7 per 100 000 persons in 2013 (95% confidence interval = 0.7-0.8).^10^ Major risk factors for MCC include advanced age, light skin complexion, immunosuppressive state, and excessive ultraviolet radiation exposure. In addition, prior studies have shown that Merkel cell polyomavirus (MCPyV) has found to be associated with development of MCC.^11-14^ MCC typically presents as a rapidly growing, painless, firm, nontender, flesh-colored or bluish-red, intracutaneous nodule without ulceration and crusting. Primary tumors are most commonly identified on sun-exposed areas such as head and neck (43%), upper limbs and shoulder (24%), and lower limbs and hip (15%).^15^

Histologically, MCC is hard to differentiate from other poorly differentiated, round, blue cell tumors, requiring hematoxylin and eosin as well as immunohistochemical stains. MCC cells usually have large basophilic nuclei with powdery dispersed chromatin and inconspicuous nucleoli, and minimal cytoplasm. Occasionally, it may show single-cell necrosis, frequent mitoses, and invasion to epidermal, lymphovascular, and perineural structures. MCC cells have both epithelial and neuroendocrine cell features. They express epithelial markers such as AE1/AE3, CAM 5.2, pancytokeratin, epithelial membrane antigen, and Ber-EP4 and possibly stain different neuroendocrine markers, including chromogranin, synaptophysin, calcitonin, vasoactive intestinal peptide, and somatostatin receptor.^16^ MCC can be differentiated by being stained with low-molecular-weight cytokeratins (eg, CK20, CK5/6) from other undifferentiated tumors (Table 1). MCC stains positively for low-molecular-weight CK20 with a characteristic paranuclear dot-like positivity.

**Table 1.** Immunocytochemical Differential Diagnosis of Small, Round, Blue Cell Tumors.

Tumor Type	CK7	CK20	TTF 1	S-100	NSE
Merkel cell carcinoma	Negative	Positive	Negative	Negative	Positive
Melanoma	Negative	Negative	Negative	Positive	Positive
Small cell carcinoma of lung	Positive	Negative	Positive	Negative	Positive

Abbreviations: CK7, cytokeratin 7; CK20, cytokeratin 20; TTF1, thyroid transcription factor 1; NSE, neuron-specific enolase.

After definitive diagnosis is made, nodal involvement and presence of metastasis needs to be determined. Sentinel lymph node biopsy may help with diagnosing occult disease in patients who do not have evidence of regional lymph node or distant metastasis. Regional lymph node involvement is the most important predictor of survival for MCC. However, other clinical and pathologic features such as intratumoral CD8+ lymphocyte infiltration, presence of the MCPyV large tumor antigen, and expression of the retinoblastoma protein are associated with good prognosis. But on the other hand, expression of p63 and antibodies against MCPyV oncoproteins (large and small tumor antigens) are associated with poor prognosis and high tumor burden.

Treatment of MCC initially involves local excision of the primary lesion with 1 to 2 cm margins in patients without regional lymphatic or distant metastases. RT is considered if wide excision is not applicable. If the primary tumor possesses high-risk features for local recurrence such as ≥1 cm in maximum dimension, positive or limited surgical resection margins, lymphovascular invasion, or an immunocompromised host, adjuvant RT following surgery can be applied. If sentinel lymph node biopsy shows lymph node metastasis, appropriate imaging studies with careful evaluation are indicated. Nodal dissection and RT are recommended to patients without distant metastasis. If metastatic disease is confirmed, treatment with a programmed cell death ligand 1 (PD-L1)-blocking agent (avelumab) or programmed cell death protein 1 (PD-1) inhibitor–based immunotherapy (pembrolizumab, nivolumab) should be used as a first-line treatment option.^17-19^ Systemic chemotherapy with platinum (cisplatin, carboplatin) plus etoposide may provide benefit to patients who do not respond to PD-1/PD-L1 inhibitor–based immunotherapy. In the JAVELIN Merkel 200 trial, efficiency of avelumab was investigated on patients with metastatic MCC. The patients reported a better experience and less side effects such as lack of energy, fatigue, limited physical activity, nausea, and decreased appetite with avelumab rather than they did with chemotherapy. However, this study did not include patients with HIV. In the future, further studies will yield advancements of this rare but aggressive neuroendocrine skin cancer.
